# Identification of a small thrombus in the left ventricle identified on iodine maps derived from dual-source photon-counting detector CT

**DOI:** 10.1016/j.radcr.2024.01.020

**Published:** 2024-01-16

**Authors:** Masaya Kisohara, Nobuo Kitera, Toshihide Itoh, Kazuma Murai, Akio Hiwatashi, Tatsuya Kawai

**Affiliations:** a Department of Radiology, Nagoya City University Graduate School of Medical Sciences, Nagoya, Japan; b Central Radiology Division, Nagoya City University Hospital, Nagoya, Japan; c CT-Research and Collaboration, Siemens Healthineers, Tokyo, Japan

**Keywords:** Left ventricular thrombus, Iodine maps, Coronary CT angiography, Transthoracic echocardiographyce

## Abstract

Transthoracic echocardiography is the main imaging modality to diagnose left ventricular thrombus (LVT), but its efficacy in certain cases is suboptimal. We report a patient in whom an LVT, initially unidentified by transthoracic echocardiography, was successfully diagnosed with iodine maps derived from dual-source photon-counting detector CT (DS-PCD-CT). The 64-year-old male was admitted to our institution following myocardial infarction. Although TTE failed to detect this small LVT, iodine maps derived from CT angiography (which was conducted to evaluate the coronary artery stenosis) revealed its presence. Iodine maps derived from DS-PCD-CT collecting data with high temporal resolution are beneficial to diagnose LVTs.

## Introduction

A left ventricular thrombus (LVT) forms within the ventricle adjacent to hypokinetic or akinetic myocardium [1]. LVT etiological factors include takotsubo cardiomyopathy and ventricular aneurysm [2]. The established diagnostic standard for identifying LVTs is transthoracic echocardiography (TTE) [[1], [2]]. LVTs are diagnosed by detecting structures displaying unimpeded movement distinct from the endocardium [1]. However, accurate diagnoses can be challenging because of the visual similarity of the ventricle's speckle and the LVT's speckle, particularly when the LVT is adherent to the ventricular wall [3]. Delayed gadolinium contrast enhancement in cardiac MRI [4], characteristic CT attenuation [5], and iodine maps derived from dual-energy CT [6] have been proposed as methods to diagnose LVTs.

Cardiac CT is prone to motion artifacts arising from myocardial contractions. A shorter rotation time of the X-ray tube leads to improved cardiac CT image quality [7]. Dual-source CT, known for its high temporal resolution, thus plays a significant role in enhancing cardiac images' quality. Photon-counting detector CT (PCD-CT) has superior spatial resolution and significantly reduced noise compared to energy-integrated detector CT (EID-CT) [[8], [9]] because photon-counting detectors do not require septa to mitigate the scattering of electrical noise [10]. Dual-source PCD-CT (DS-PCD-CT), which is now available for clinical use, can collect data with high spatial and temporal resolution. The significant potential of DS-PCD-CT for noninvasively assessing coronary artery stenosis is attributable to its high-resolution images with minimal motion artifacts [11], [12], [13], [14].

PCD-CT also enhances the discrimination of the iodine component by exploiting the direct conversion of X-ray photons into an electrical signal. This approach stands in contrast to EID-CT, which requires the use of 2 different tube voltages solely for generating virtual monoenergetic images, limiting the precision of iodine extraction [15].

Although it has been reported that DS-PCD-CT's high temporal resolution is useful for evaluating coronary arteries [16], we have found no report concerning the usefulness of iodine maps derived from DS-PCD-CT. We report the case of an LVT diagnosed by DS-PCD-CT.

## Case

The patient was a 64-year-old male admitted to our institution's neurology department for comprehensive examinations related to muscular disease. Electrocardiography and echocardiography showed abnormal findings including dyskinesis of the left-ventricular-wall motion, and the patient was referred to our cardiology department. The ECG showed a QS pattern, leading to the diagnosis of subacute myocardial infarction. There were no subjective symptoms.

**Fig. 1:**
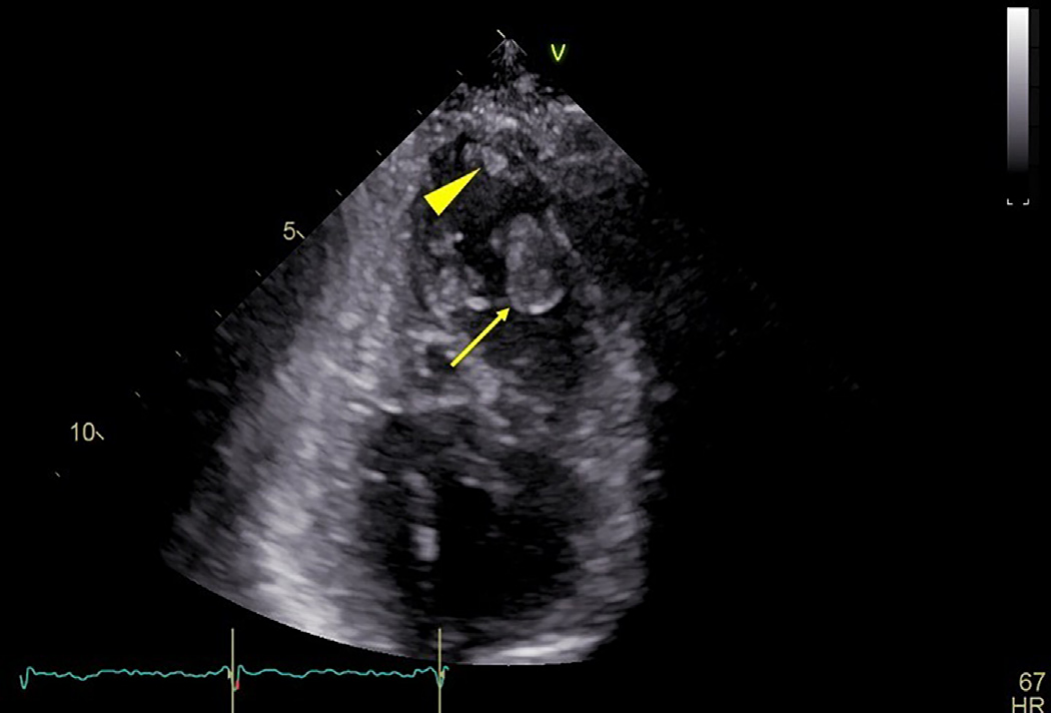
A 64-year-old man with left ventricular thrombi, detected by 2-chamber transthoracic echocardiography (*arrow* and *arrowhead*). The larger thrombus (*arrow*) was easy to identify because of its floating movement. However, the small thrombus (*arrowhead*) adherent to the myocardium was not detected at the time of examination.

An LVT was detected as a free-moving structure in the left ventricular aneurysm on TTE (Fig. 1, arrow). For the assessment of the LVT and coronary arteries, coronary CT angiography (CCTA) and delayed-phase contrast-enhanced CT were conducted using a gated spiral scan on a DS-PCD-CT system (NAEOTOM Alpha, Siemens Healthineers, Forchheim, Germany). These were performed after an administration of iodinated contrast media (Iopamidol-370, Bayer, Nordrhein-Westfalen, Germany) through the right antecubital vein at 25 mgI/kg/s, followed by a saline flush and a test injection method. The CCTA reconstruction conditions were as follows: 0.2 mm slice thickness, Bv44 kernel, iterative-reconstruction (IR) strength at 4, and an optimal cardiac phase obtained at 79% of the R-R interval. The delayed-phase contrast-enhanced images, captured at 50-second postcontrast media injection, were obtained through a gated high-pitch spiral scan. No additional contrast agent was administered for this scanning. The reconstruction conditions were 0.4 mm slice thickness, Bv44 kernel, and IR strength at 3.

**Fig. 2:**
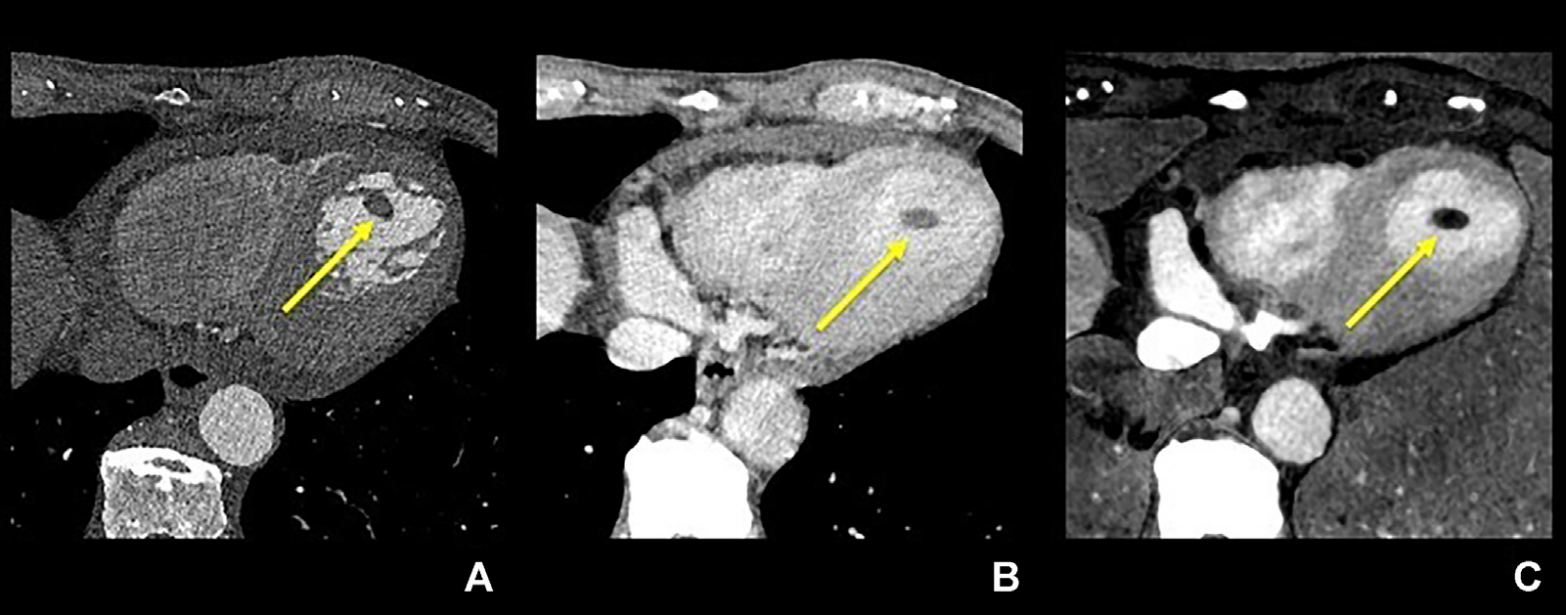
The larger thrombus is visualized on axial images scanned with photon-counting-detector CT: the coronary arterial phase (A), the delayed phase scanned 50 sec after a contrast media injection (B), and the iodine map made from the delayed phase (C) Note that the iodine map better visualizes the thrombus than other 2 images because of the higher contrast between the thrombus and myocardium.

Iodine maps derived from the delayed-phase contrast-enhanced images were obtained. The iodine maps' reconstruction conditions were 1.0 mm slice thickness, Qr36 kernel, and IR strength at 4. We reconstructed all images with a 144  ×  144-mm^2^ field of view and a matrix size of 512  ×  512 pixels. The CT dose index (CTDI_vol_)values of the CCTA and the delayed-phase scanning were 33.5 and 6.0 mGy, respectively. The CCTA showed a complete occlusion of the left anterior descending branch and a 16  ×  10  ×  7-mm^3^ nodule-like structure similar to the thrombus noted on TTE in the left ventricular aneurysm (Fig. 2A). It was easily diagnosed as a thrombus, primarily because it exhibited a lack of contrast enhancement (unlike the surrounding myocardium), as evident in the delayed-phase images (Fig. 2B). Moreover, its anatomical separation from the left ventricle supported the diagnosis. Iodine maps confirmed the absence of iodine uptake in this structure (Fig. 2C).

**Fig. 3:**
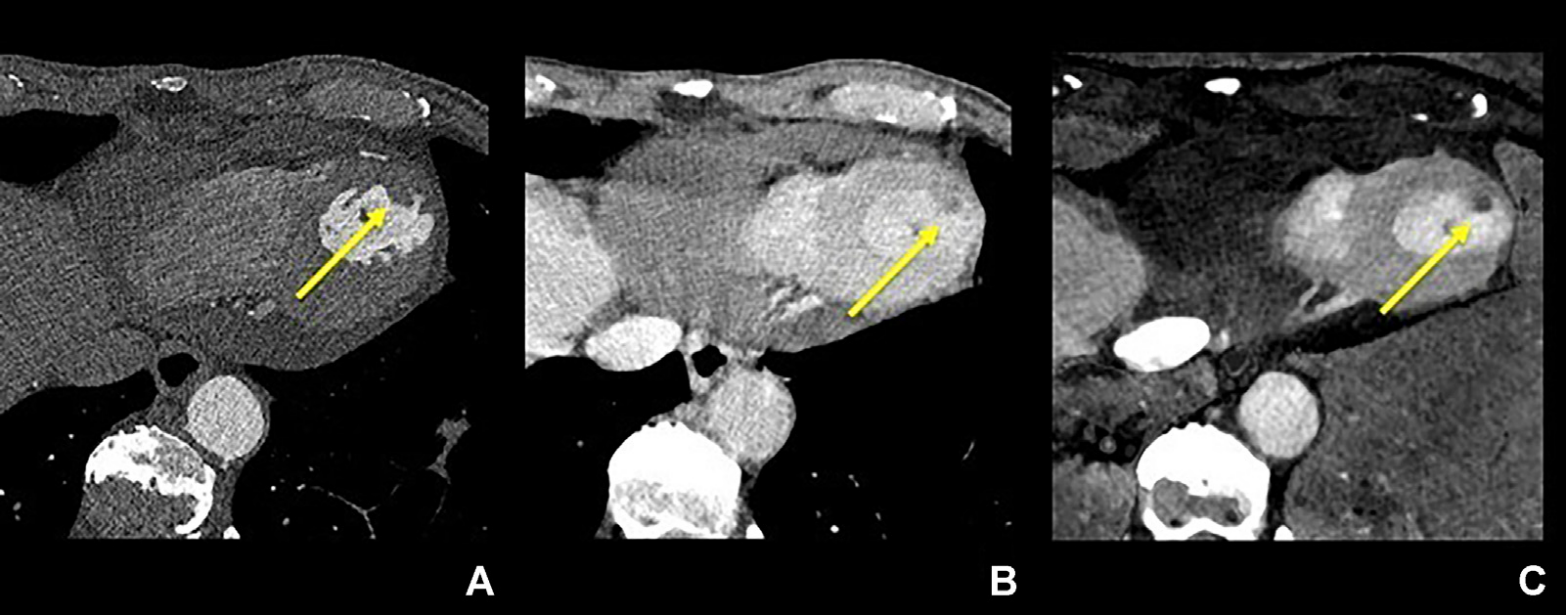
The smaller thrombus adherent to the myocardium on axial images scanned with photon-counting-detector CT: the coronary arterial phase (A), the delayed phase scanned 50 seconds after a contrast media injection (B), and the iodine map made from the delayed phase (C). Note that the iodine map also better visualizes the thrombus than other 2 images because of the higher contrast between the thrombus and myocardium.

**Fig. 4:**
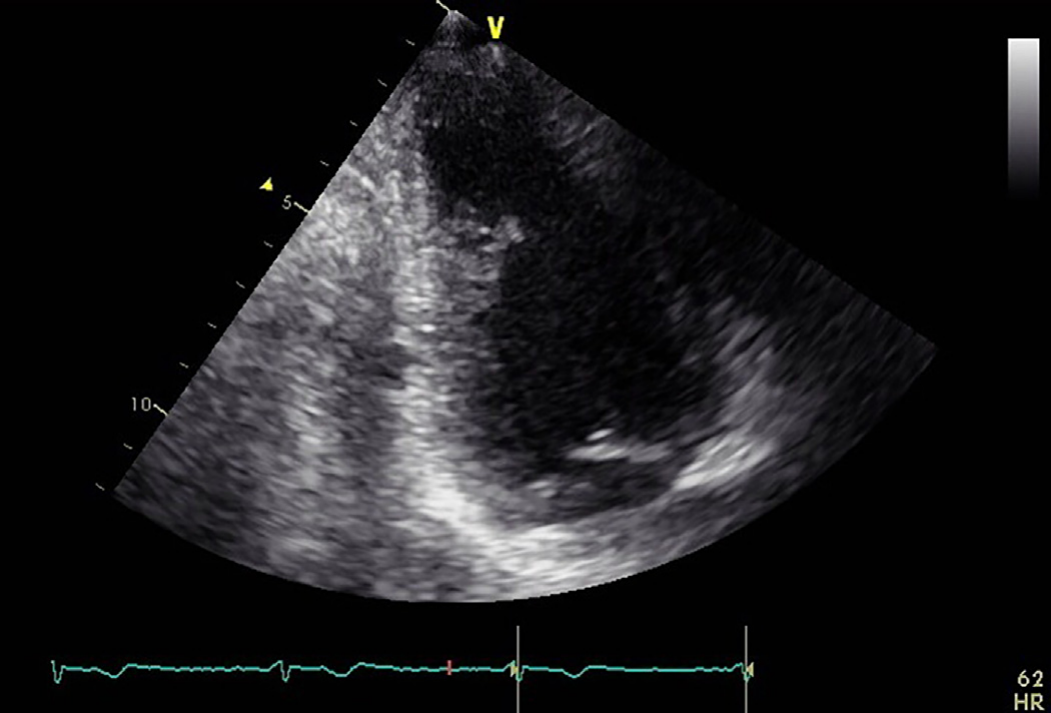
The patient's follow-up transthoracic echocardiography (TTE) showed no remaining thrombi, further supporting the diagnosis after anticoagulant treatment.

Another structure measuring 4  ×  4  ×  4 mm^3^ and visually similar to myocardial tissue was identified by CCTA (Fig. 3A). It exhibited a slight contrast defect on the delayed-phase image (Fig. 3B). This structure was clearly delineated as an iodine-free area on iodine maps, suggesting that it too was an LVT (Fig. 3C). Among all of the images, the iodine maps exhibited the highest level of contrast between the 2 LVTs and the myocardium. The latter LVT was only identified retrospectively on the initial TTE as the similar structure depicted on the CT images (Fig. 1, arrowhead). After anticoagulant treatment (17 days), follow-up TTE revealed no remaining thrombi, further supporting the diagnosis (Fig. 4). The patient was discharged from the hospital without complications.

## Discussion

Our patient's case demonstrates the successful diagnosis of LVTs with the use of DS-PCD-CT, capitalizing on its state-of-the-art X-ray photon-counting detector technology, which offers high spatial resolution and precise iodine detection. The patient presented 2 LVTs; a sizable thrombus and a small thrombus adhering to the left ventricular myocardium at the apex. Although the larger LVT moved freely within the left ventricular aneurysm, making it easily diagnosable with TTE alone, the smaller LVT proved more challenging to diagnose even in retrospect with TTE. Conversely, the smaller LVT was easily identified due to its poor iodine uptake, discernible on the iodine maps derived from DS-PCD-CT, which provided a remarkable temporal resolution of 66 ms for data acquisition.

Bittencourt et al. [5] reported the detection of LVTs on iodine maps in 2012. They noted that diagnosing LVTs can be facilitated by the contrast observed between the myocardium and the thrombus on iodine maps, corroborating our findings. To obtain iodine maps, the temporal resolution of dual-source EID-CT (DS-EID-CT) must be halved, because DS-EID-CT requires 180°of data per detector to produce dual-energy data [17]. DS-EID-CT provides temporal resolution that is essentially equivalent to that of a single-source system. The exceptional spatial registration between low- and high-energy images, facilitated by the high temporal resolution of DS-PCD-CT, improves the precision of iodine maps, making them more resilient to motion artifacts compared to DS-EID-CT imaging [18]. The advantages of DS-PCD-CT, particularly its high temporal resolution and motion artifact robustness, significantly contributed to the detection of our patient's smaller LVT. In his case, the ability to diagnose the small thrombus adherent to myocardium was attributable to the iodine maps derived from DS-PCD-CT's precise data-collection capabilities.

This case underscores the significant clinical potential of DS-PCD-CT for diagnosing cardiac lesions based on its advanced X-ray photon-counting detector technology, which enables high spatial resolution and accurate iodine detection, as demonstrated herein by its successful diagnosis of LVTs. A further accumulation of cases using DS-EID-CT and DS-PCD-CT may provide insights into the minimum detectable size of thrombi.

## Patient consent

We explained the use of the specimens for clinical research to the individual patients and obtained their consent.
